# Prevalence, incidence and management of atopic dermatitis in Australian general practice using routinely collected data from MedicineInsight

**DOI:** 10.1111/ajd.13268

**Published:** 2020-03-15

**Authors:** Kendal Chidwick, Doreen Busingye, Allan Pollack, Rawa Osman, Jeannie Yoo, Suzanne Blogg, Diana Rubel, Saxon Smith

**Affiliations:** ^1^ NPS MedicineWise Sydney New South Wales Australia; ^2^ Australian National University Canberra Australian Capital Territory Australia; ^3^ Woden Dermatology Canberra Australian Capital Territory Australia; ^4^ The Canberra Hospital Canberra Australian Capital Territory Australia; ^5^ Northern Clinical School Sydney Medical School University of Sydney Sydney New South Wales Australia; ^6^ The Dermatology and Skin Cancer Centre St Leonards New South Wales Australia; ^7^ Department of Dermatology Royal North Shore Hospital St Leonards New South Wales Australia

**Keywords:** atopic dermatitis, Australia, eczema, general practice, incidence, management, MedicineInsight, prevalence, therapies

## Abstract

**Background/Objectives:**

The prevalence of atopic dermatitis (AD) has increased significantly in industrialised countries in recent decades but data about the incidence or prevalence of AD in Australia are sparse. We aimed to determine the prevalence and incidence of AD among patients seen in Australian general practice and the use of specified medicines.

**Methods:**

This was a cross‐sectional study of 2.1 million patients attending 494 general practices in the MedicineInsight program from 1 January 2017 to 31 December 2018. We assessed the prevalence (lifetime and current), incidence, management and severity of AD.

**Results:**

The lifetime (ever diagnosed) prevalence of AD in this general practice population was 16.4% and was greater in females (17.3%) than males (15.3%). One in five patients with AD were classified as having moderate‐to‐severe disease. Prevalence over the last two years was 6.3%. The incidence of AD in 2018 was 2.0% and was greater in females (2.2%) and for patients aged 0–4 years (3.9%). Patients with AD had an increased risk of insomnia, anxiety and depression, compared to those with no recorded AD. For AD patients, topical corticosteroids were the most commonly prescribed AD medication (36.5%) and topical calcineurin inhibitors the least (0.1%), with systemic corticosteroids (15.6%) more commonly prescribed than other immunosuppressants (0.9%).

**Conclusions:**

Our findings provide important insights into the epidemiology of AD and its management in Australian general practice. This information is likely to be useful in planning effective interventions to support GPs in the optimal management of patients with AD.

## Introduction

Atopic dermatitis, also called eczema, is a chronic, relapsing, pruritic, inflammatory skin disease that occurs more frequently in children than adults.^1^ The prevalence of eczema varies widely between populations and countries^2^, ^3^ and is said to have doubled or tripled in industrialised countries in recent decades.^4^, ^5^ The population prevalence of eczema in Australia was estimated to be 16% in 4‐year‐olds (2017)^6^ and 20.3% in 1‐year‐olds (2013).^7^

Atopic dermatitis has substantial effects on the psychosocial well‐being and quality of life of patients,^8^, ^9^ who often have comorbidities such as asthma, allergic rhinoconjunctivitis, food allergies and mental illnesses. Evidence shows an increased prevalence of depression, anxiety and conduct disorder in children severely affected by atopic dermatitis.^10^ Children and adolescents with atopic dermatitis have approximately 50% greater risk of attention deficit hyperactivity disorder, which is likely a result of sleep disturbance.^11^ Adults with atopic dermatitis are three times as likely to have depression compared with healthy individuals,^12^ and the prevalence of suicidal ideation exceeds 20%.^13^

Atopic dermatitis also places a tremendous financial burden on patients, their families, and society, through direct medical costs and decreased productivity.^14^, ^15^ Severe atopic dermatitis costs over AUS$ 6000 annually per child in direct medical, hospital and treatment costs, as well as time off work and distress for caregivers and the family.^16^

Mild atopic dermatitis is typically managed in primary care by avoiding irritants and disease triggers and by using emollients (moisturisers) and standard topical anti‐inflammatory therapies such as corticosteroids, calcineurin inhibitors and phosphodiesterase type 4 inhibitors.^17^ Other therapies used to manage atopic dermatitis include systemic agents such as corticosteroids, biologics and other immunosuppressants; phototherapy; and other agents (such as antihistamines and antibacterial medicines). The Australian management consensus recommends referral to a dermatologist or immunologist if atopic dermatitis is not responsive to standard treatment, causes significant distress and interferes with sleep, school or work, an allergy is suspected, or there are recurrent infections.^17^

Although the burden of atopic dermatitis is said to be increasing in Australia,^3^, ^18^ national information about the prevalence and particularly the incidence of atopic dermatitis is limited. Using routinely collected general practice data from the MedicineInsight program, this study aimed to 1) determine the prevalence and incidence of atopic dermatitis among patients seen in Australian general practice; 2) describe the use of medicines for the management of patients with atopic dermatitis; and 3) classify patients with atopic dermatitis by disease severity and assess relevant comorbidities.

## Methods

### Study design and data source

We conducted a descriptive study using MedicineInsight, a national general practice data program developed and managed by NPS MedicineWise with funding support from the Australian Government Department of Health.^19^ MedicineInsight extracts and collates longitudinal, de‐identified patient health records, including demographics, encounters (excluding progress notes), diagnoses, prescriptions and pathology tests from MedicalDirector and Best Practice clinical information systems. On 1 July 2019, MedicineInsight included records for about 3.5 million regular patients (approximately 15% of the Australian population) from more than 5000 GPs in 715 general practices across Australia. When compared with Medicare Benefits Schedule data, the characteristics of current regular MedicineInsight patients are broadly comparable to those of patients who visited a GP in 2016–17.^19^

### Study participants

We included patients who had valid information for age and gender and were regular attenders (at least three consultations between 1 January 2017 and 31 December 2018 in accordance with the Royal Australian College of General Practitioners’ (RACGP) definition of an active patient) at a practice that met data quality requirements (described elsewhere).^19^

A sub‐cohort was used to estimate the incidence of atopic dermatitis and included patients from the main study population (Figure S1) who met additional inclusion criteria: did not have a diagnosis of atopic dermatitis recorded prior to 1 January 2018 (historical diagnosis) and had at least 12 months of follow‐up (attendance) at the practice prior to 1 January 2018.

### Atopic dermatitis diagnoses

**Figure 1:**
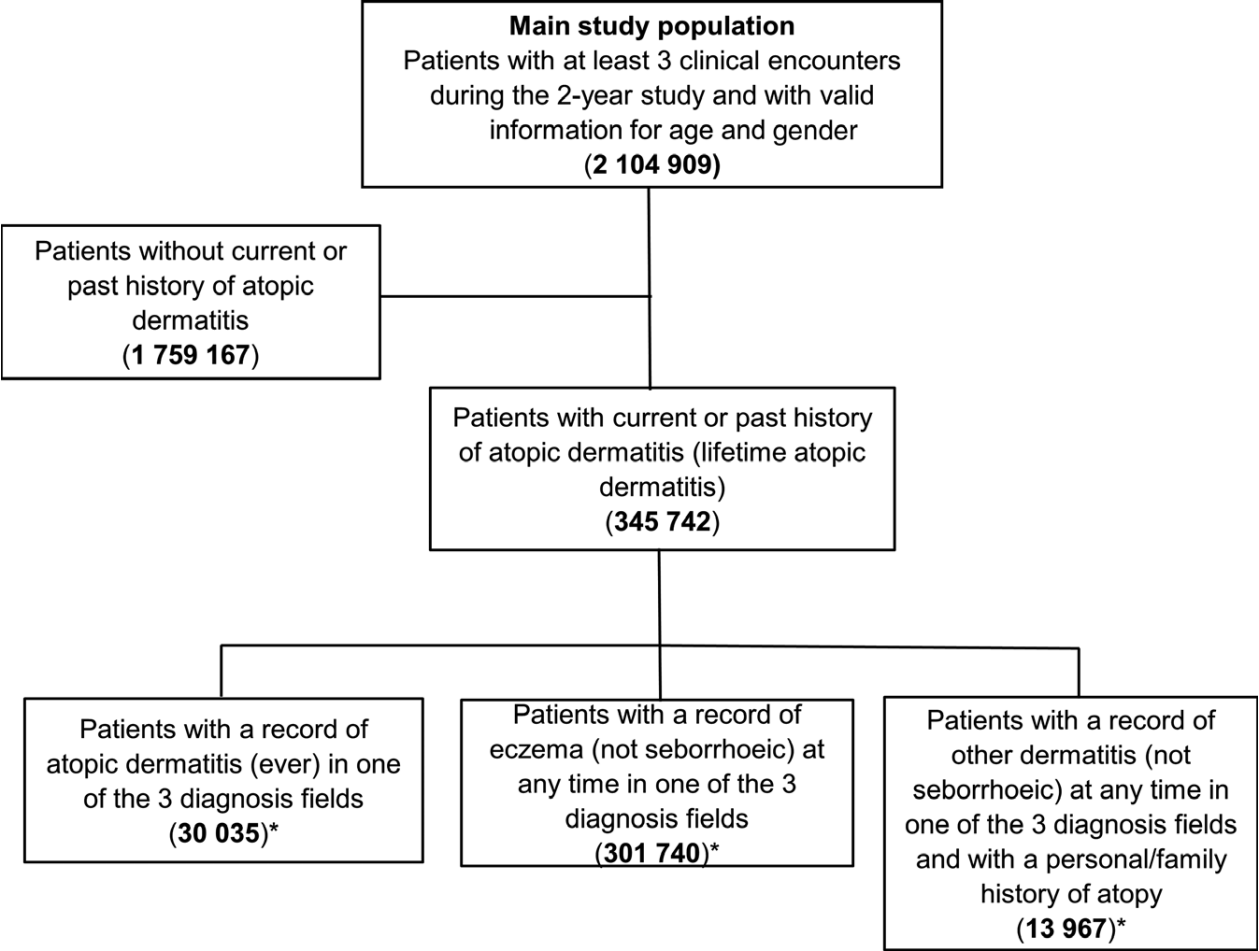
Flow chart for selection of the main study population and atopic dermatitis prevalent population. ^*^Patients in these groups are mutually exclusive, with the order of priority being ‘atopic dermatitis’, then ‘eczema’ and then ‘dermatitis (other)’ plus personal/family history of atopy. That is, patients with recorded ‘atopic dermatitis’ are counted first, then those with recorded ‘eczema’ (and no recorded ‘atopic dermatitis’) and then the rest.

We developed condition coding algorithms using information from the diagnosis (medical history), reason for encounter and reason for prescription fields, and included both coded and free‐text data. Patients were defined as having atopic dermatitis if they had a relevant term recorded at any time (ever) from their earliest record until the end of the study: ‘atopic dermatitis’; or ‘eczema’ (except seborrhoeic); or ‘other dermatitis (not seborrhoeic) and a personal/family history of atopy (ever)’ (Fig. 1). ‘Atopic conditions’ included atopy, atopic dermatitis, eczema, asthma, allergic rhinoconjunctivitis and food allergies including milk (dairy), eggs, nuts (tree and peanuts), sesame, fish and seafood. A full list of included terms is in Table S1.

### Management of atopic dermatitis

We described the type of specified atopic dermatitis medications prescribed and eosinophil count tests, among patients with atopic dermatitis. While not a diagnostic test, an elevated eosinophil count can support the diagnosis of atopy.^20^

Medicine groups assessed included topical corticosteroids (TCS), topical calcineurin inhibitors (TCI), systemic corticosteroids, systemic immunosuppressants (ciclosporin, methotrexate, azathioprine or mycophenolate) and antibacterial medicines (mupirocin, dicloxacillin, flucloxacillin, cefalexin, clindamycin [non‐topical form], trimethoprim/sulfamethoxazole or erythromycin). The classification of topical corticosteroids by potency is described in Table S2.

MedicineInsight prescribing information includes whether the prescription is marked as eligible for Australian Government subsidy (under the Pharmaceutical Benefits Scheme [PBS] or the Repatriation Pharmaceutical Benefits Scheme [RPBS], which is available to specified war veterans and their families) or not eligible, namely ‘private’. Medicines were identified using the Anatomical Therapeutic Chemical Classification System code, ‘medicine active ingredient’, ‘medicine name’ and, where required, the route of administration and strength. A patient was defined as having one of the assessed medicine groups if they had at least one recorded relevant prescription in the 2‐year study period. Since it is not mandatory for prescribers to record a reason for prescription, this field is often incomplete. Therefore, some of the specified atopic dermatitis medicine prescriptions counted for the patients with atopic dermatitis may have been for indications other than atopic dermatitis.

A patient was defined as having an eosinophil count test result if they had at least one properly recorded test result in the 2‐year study period – identified by the relevant Logical Observation Identifiers Names and Codes. Eosinophilia was defined as at least one eosinophil count test result where the upper limit value exceeded the laboratory’s normal reference range for that patient.

### Classification of atopic dermatitis severity

There is no validated measure of atopic dermatitis severity for routinely collected clinical data. Data, such as objective signs and subjective symptoms on which validated measures such as the SCORing Atopic Dermatitis index are based, are not available in MedicineInsight, and this presents challenges in classifying patients according to severity. For this analysis, patients were classified into two categories – ‘mild’ or ‘moderate‐to‐severe' – in accordance with the Australian consensus recommendations for management of atopic dermatitis.^17^ A record of systemic therapies for atopic dermatitis or a referral to a specialist were used as indicators of disease severity in this study.

Patients with atopic dermatitis were classified as having ‘moderate‐to‐severe’ atopic dermatitis if they had either a prescription for systemic immunosuppressants or corticosteroids or if they had a referral to a dermatologist/immunologist/allergist, recorded during the study period. Otherwise, they were classified as having ‘mild’ atopic dermatitis.

### Outcomes

#### Prevalence

Atopic dermatitis is a chronic relapsing condition where some patients outgrow the disease. Because it is difficult to define current atopic dermatitis in routinely collected data, both lifetime and current prevalence estimates were assessed.

Lifetime prevalence was estimated as the proportion of the main study population who had current, or a past history of, atopic dermatitis (ever‐recorded diagnosis).

Current prevalence was estimated as the proportion of the main study population who had a diagnosis of atopic dermatitis recorded during the 2‐year study period.

#### Incidence

The incidence of atopic dermatitis was estimated as the proportion of the sub‐analysis cohort (Figure S1) who had a new diagnosis of atopic dermatitis recorded for the first time during 2018.

### Covariates

Socio‐demographic characteristics included age (based on year of birth), gender, state/territory, Socio‐Economic Indexes for Areas (SEIFA) and remoteness. State/territory, remoteness and SEIFA were based on patients’ residential postcodes. Remoteness was determined in accordance with the ABS geographical framework ‘Remoteness Areas’.^21^ SEIFA was determined according to the Australian Bureau of Statistics Index of Relative Socio‐Economic Advantage and Disadvantage (IRSAD).^22^ IRSAD is an indicator of relative economic and social advantage/disadvantage position within an area compared with the rest of the country.

We assessed the presence of relevant comorbidities including insomnia, anxiety and depression (see Table S1 for detailed clinical definitions).

### Statistical analysis

We used descriptive statistics including frequencies, proportions and relative risks adjusted for confounding factors, age and gender. 95% confidence intervals (CIs) were adjusted for clustering by practice site. Non‐overlap of 95% CIs was used to determine significant differences between groups.^23^ Severity of atopic dermatitis and medications were assessed among patients with an ever‐recorded diagnosis of atopic dermatitis (‘lifetime atopic dermatitis’). Data management and analyses were conducted using SAS version 9.4 (SAS Institute Inc., Cary, NC, USA).

### Ethics

Approval to conduct this study was granted by the MedicineInsight independent Data Governance Committee (reference number: 2018‐014). NPS MedicineWise has RACGP NREEC ethics approval (NREEC 17‐017) for the standard operation and the use of the MedicineInsight program by NPS MedicineWise.

## RESULTS

### Characteristics of the main study population

The main study population comprised 2 104 909 regular patients from 494 general practices (Fig. 1). Of this population, 56.6% were female, 63.7% resided in major cities, and the median age was 43.0 years.

### Characteristics of patients with atopic dermatitis

There were 345 742 patients with a current or historical (lifetime) diagnosis of atopic dermatitis recorded, of whom 21.4% were classified as having ‘moderate‐to‐severe' disease (data not shown). Just over one in seven patients (14.9%) with atopic dermatitis were aged below 10 years (Table 1).

**Table 1:** Socio‐demographic characteristics of patients with and without atopic dermatitis (ever) (2017–2018)

Characteristic	Patients with atopic dermatitis (ever) (*N* = 345 742)		Patients without atopic dermatitis (*N* = 1 759 167)	
	Number	% (95% CI)	Number	% (95% CI)
Gender				
Male	140 182	40.5 (40.0, 41.1)	773 875	44.0 (43.5, 44.4)
Female	205 560	59.5 (58.9, 60.0)	985 292	56.0 (55.6, 56.5)
Age group (years)				
0–4	27 111	7.8 (7.2, 8.5)	117 268	6.7 (6.4, 7.0)
5–9	24 285	7.0 (6.7, 7.3)	85 558	4.9 (4.7, 5.0)
10–14	18 382	5.3 (5.1, 5.5)	71 351	4.1 (3.9, 4.2)
15–19	17 159	5.0 (4.8, 5.2)	82 615	4.7 (4.5, 4.8)
20–24	16 648	4.8 (4.6, 5.0)	101 685	5.8 (5.4, 6.2)
25–29	15 766	4.6 (4.3, 4.8)	113 691	6.5 (6.1, 6.8)
30–34	16 375	4.7 (4.5, 5.0)	125 141	7.1 (6.8, 7.5)
35–39	17 131	4.9 (4.7, 5.2)	124 109	7.1 (6.8, 7.3)
40–44	16 859	4.9 (4.7, 5.0)	113 550	6.4 (6.3, 6.6)
45–49	19 206	5.6 (5.4, 5.7)	122 939	7.0 (6.8, 7.1)
50–54	19 106	5.5 (5.4, 5.7)	114 209	6.5 (6.4, 6.6)
55–59	21 664	6.3 (6.1, 6.5)	118 907	6.8 (6.6, 6.9)
60–64	21 297	6.2 (6.0, 6.4)	110 821	6.3 (6.1, 6.5)
65–69	22 402	6.5 (6.2, 6.7)	104 068	5.9 (5.7, 6.2)
70–74	22 531	6.5 (6.2, 6.8)	91 724	5.2 (4.9, 5.5)
75–79	18 123	5.2 (4.9, 5.5)	64 032	3.6 (3.4, 3.9)
80–84	14 492	4.2 (3.9, 4.4)	45 253	2.6 (2.4, 2.8)
85–89	9988	2.9 (2.7, 3.1)	30 266	1.7 (1.6, 1.8)
90+	7217	2.1 (1.9, 2.3)	21 980	1.3 (1.1, 1.4)
State/territory				
ACT	6084	1.8 (0.4, 3.1)	34 764	2.0 (0.4, 3.6)
NSW	133 168	38.5 (32.7, 44.3)	690 161	39.2 (33.7, 44.7)
NT	3132	0.9 (0.2, 1.6)	30 316	1.7 (0.5, 3.0)
QLD	45 073	13.0 (9.7, 16.4)	295 278	16.8 (12.9, 20.6)
SA	12 340	3.6 (1.6, 5.6)	45 387	2.6 (1.1, 4.1)
TAS	27 360	7.9 (4.5, 11.3)	103 194	5.9 (3.3, 8.4)
VIC	87 262	25.2 (19.4, 31.0)	359 929	20.5 (15.6, 25.3)
WA	31 323	9.1 (5.8, 12.3)	200 138	11.4 (7.4, 15.3)
Remoteness				
Major city	222 913	64.5 (59.1, 69.9)	1 118 700	63.6 (58.7, 68.5)
Inner regional	78 799	22.8 (18.5, 27.1)	417 392	23.7 (19.6, 27.8)
Outer regional	40 004	11.6 (7.7, 15.4)	196 000	11.1 (8.1, 14.2)
Remote/very remote	4026	1.2 (0.5, 1.8)	27 075	1.5 (0.7, 2.4)
Socio‐economic status (SEIFA IRSAD quintiles)				
1 (least advantaged)	59 038	17.1 (13.4, 20.7)	265 479	15.1 (12.2, 18.0)
2	59 526	17.2 (14.3, 20.1)	315 878	18.0 (15.2, 20.7)
3	75 964	22.0 (18.9, 25.1)	403 309	22.9 (19.8, 26.0)
4	72 125	20.9 (18.2, 23.5)	378 739	21.5 (18.8, 24.2)
5 (most advantaged)	78 951	22.8 (18.9, 26.8)	394 743	22.4 (18.9, 25.9)
Data missing	138	0.0 (0.0, 0.1)	1019	0.1 (0.0, 0.1)
Comorbid conditions				
Insomnia	46 957	13.6 (12.9, 14.3)	128 587	7.3 (7.0, 7.6)
Anxiety	70 751	20.5 (19.6, 21.3)	263 427	15.0 (14.4, 15.5)
Depression	78 083	22.6 (21.7, 23.5)	296 267	16.8 (16.2, 17.4)

CI, confidence interval; IRSAD, Index of Relative Socio‐economic Advantage and Disadvantage; SEIFA, Socio‐economic Indexes for Areas.

### Lifetime and current prevalence of atopic dermatitis

The estimated lifetime prevalence of atopic dermatitis was 16.4% (Table 2). Recorded lifetime prevalence for patients aged below 15 and those ≥65 years, and for patients resident in South Australia, Tasmania and Victoria, was greater than the overall population prevalence. It was greater for females (17.3%; 95% CI 16.4–18.1) than males (15.3%; 95% CI 14.6–16.0).

**Table 2:** Lifetime prevalence and current prevalence of atopic dermatitis (2017–2018)

Characteristic	Lifetime prevalence of atopic dermatitis (*N* = 2 104 909)	Current prevalence of atopic dermatitis (*N* = 2 104 909)
	% (95% CI)	% (95% CI)
Overall	16.4 (15.7, 17.2)	6.3 (6.0, 6.6)
Gender		
Male	15.3 (14.6, 16.0)	6.1 (5.8, 6.4)
Female	17.3 (16.5, 18.1)	6.5 (6.2, 6.8)
Age group (years)		
0–4	18.8 (17.9, 19.7)	13.8 (13.1, 14.5)
5–9	22.1 (21.1, 23.1)	8.3 (7.9, 8.7)
10–14	20.5 (19.4, 21.6)	6.5 (6.2, 6.8)
15–19	17.2 (16.2, 18.2)	5.8 (5.5, 6.1)
20–24	14.1 (13.1, 15.0)	5.6 (5.3, 5.9)
25–29	12.2 (11.5, 12.9)	5.2 (4.9, 5.5)
30–34	11.6 (10.9, 12.2)	5.0 (4.8, 5.3)
35–39	12.1 (11.5, 12.7)	4.9 (4.7, 5.2)
40–44	12.9 (12.2, 13.6)	4.8 (4.5, 5.1)
45–49	13.5 (12.8, 14.2)	4.6 (4.3, 4.9)
50–54	14.3 (13.6, 15.1)	4.9 (4.6, 5.2)
55–59	15.4 (14.6, 16.2)	5.3 (4.9, 5.6)
60–64	16.1 (15.3, 16.9)	5.4 (5.1, 5.8)
65–69	17.7 (16.8, 18.7)	5.9 (5.5, 6.3)
70–74	19.7 (18.7, 20.8)	6.5 (6.1, 7.0)
75–79	22.1 (20.9, 23.2)	7.5 (6.9, 8.0)
80–84	24.3 (23.0, 25.6)	8.0 (7.4, 8.6)
85–89	24.8 (23.5, 26.2)	7.6 (7.0, 8.2)
90+	24.7 (23.3, 26.1)	7.1 (6.5, 7.7)
State/territory		
ACT	14.9 (11.3, 18.5)	7.0 (4.9, 9.0)
NSW	16.2 (15.0, 17.4)	6.2 (5.7, 6.7)
NT	9.4 (7.9, 10.8)	4.0 (2.9, 5.1)
QLD	13.2 (11.9, 14.5)	5.3 (4.7, 5.8)
SA	21.4 (17.6, 25.2)	6.5 (5.6, 7.5)
TAS	21.0 (19.0, 22.9)	7.0 (5.9, 8.0)
VIC	19.5 (17.8, 21.2)	7.6 (7.0, 8.3)
WA	13.5 (11.7, 15.3)	5.6 (5.0, 6.3)
Remoteness		
Major city	16.6 (15.6, 17.6)	6.6 (6.3, 7.0)
Inner regional	15.9 (14.7, 17.1)	5.7 (5.2, 6.1)
Outer regional	16.9 (14.9, 19.0)	6.0 (5.1, 6.8)
Remote/very remote	12.9 (9.8, 16.1)	5.2 (4.1, 6.3)
Socio‐economic status (SEIFA IRSAD quintiles)		
1 (least advantaged)	18.2 (16.6, 19.8)	6.6 (5.8, 7.3)
2	15.9 (14.8, 16.9)	6.1 (5.6, 6.6)
3	15.8 (14.7, 17.0)	6.2 (5.8, 6.6)
4	16.0 (14.9, 17.1)	6.4 (5.9, 6.9)
5 (most advantaged)	16.7 (15.4, 17.9)	6.3 (5.9, 6.8)

CI, confidence interval; IRSAD, Index of Relative Socio‐economic Advantage and Disadvantage; SEIFA, Socio‐economic Indexes for Areas.

The estimated current prevalence of atopic dermatitis was 6.3% (Table 2). Recorded current prevalence was greater for patients aged 0–4 years (13.8%) than for other age groups; greater for residents of major cities than inner regional and remote areas; and greater for residents of Victoria than Northern Territory, Queensland, Western Australia and New South Wales. The socio‐demographic characteristics of patients with ‘current atopic dermatitis’ were similar to those with ‘lifetime atopic dermatitis’ (Table S3).

### Incidence of atopic dermatitis

**Figure 2:**
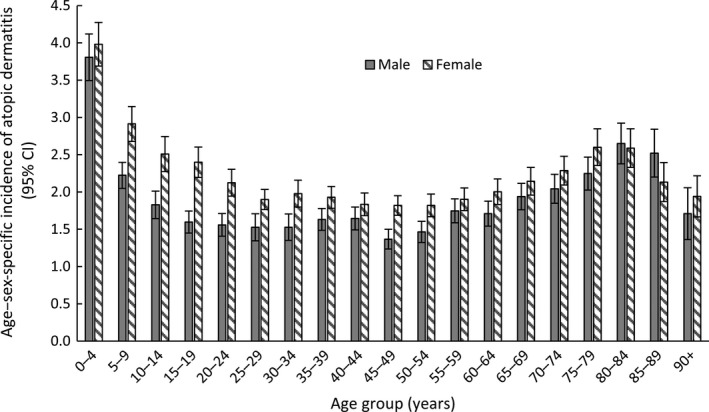
Age–sex‐specific incidence of atopic dermatitis in 2018.

Of the 1.35 million patients in the sub‐analysis cohort, 27 414 were diagnosed with atopic dermatitis during 2018, giving an estimated incidence of 2.0% (Table 3). Incidence was greater for patients aged 0–4 years (3.9%) than for other age groups, and greater for females (2.2%; 95% CI 2.1–2.3) than males (1.9%; 95% CI 1.8–2.0) (Table 3). The incidence of atopic dermatitis was similar between sexes for those aged 0–4 years, but higher in female than male patients aged 5–39 and 45–54 years (Fig. 2). The socio‐demographic characteristics of the sub‐analysis cohort and patients with incident atopic dermatitis are presented in Table S4.

**Table 3:** Incidence of atopic dermatitis in 2018

Characteristic	Sub‐analysis cohort (*N* = 1 349 224)	Patients with incident atopic dermatitis	
		Number	% (95% CI)
Overall	1 349 224	27 414	2.0 (1.9, 2.1)
Gender			
Male	592 491	11 079	1.9 (1.8, 2.0)
Female	756 733	16 335	2.2 (2.1, 2.3)
Age group (years)			
0–4	51 828	2017	3.9 (3.6, 4.2)
5–9	67 623	1727	2.5 (2.4, 2.7)
10–14	58 001	1250	2.2 (2.0, 2.3)
15–19	64 222	1311	2.0 (1.9, 2.2)
20–24	68 658	1309	1.9 (1.8, 2.0)
25–29	72 959	1288	1.8 (1.6, 1.9)
30–34	84 554	1535	1.8 (1.7, 2.0)
35–39	90 546	1642	1.8 (1.7, 1.9)
40–44	88 823	1560	1.8 (1.6, 1.9)
45–49	100 092	1628	1.6 (1.5, 1.7)
50–54	95 017	1581	1.7 (1.5, 1.8)
55–59	100 594	1842	1.8 (1.7, 2.0)
60–64	94 523	1765	1.9 (1.7, 2.0)
65–69	89 736	1837	2.1 (1.9, 2.2)
70–74	80 413	1744	2.2 (2.0, 2.3)
75–79	57 053	1388	2.4 (2.2, 2.6)
80–84	40 061	1048	2.6 (2.4, 2.8)
85–89	26 156	600	2.3 (2.1, 2.5)
90+	18 365	342	1.9 (1.6, 2.1)

CI, confidence interval.

### Comorbid conditions

Among 345 742 patients who had ever had atopic dermatitis, 46 957 (13.6%) had a record of insomnia, 70 751 (20.5%) anxiety and 78 083 (22.6%) depression (Table 1). After adjusting for age and gender, having atopic dermatitis increased the risk of insomnia, anxiety and depression by 79%, 44% and 41%, respectively, compared with the general patient population without atopic dermatitis (data not shown).

### Management of atopic dermatitis

Topical corticosteroids were the most commonly prescribed group of medicines with one in three patients with atopic dermatitis having at least one prescription for a topical corticosteroid during the study period (Table 4). The most commonly prescribed class of topical corticosteroids was potent TCS (22.5%), followed by moderate (11.5%), mild (7.5%) and very potent TCS (0.8%). The least prescribed group of medicines were topical calcineurin inhibitors (0.1%). Among patients with ‘lifetime atopic dermatitis’, 15.6% had at least one prescription for systemic corticosteroids, 0.9% had at least one prescription for other systemic immunosuppressants; 40.3% did not have a recorded prescription for any of the specified medicine groups (topical corticosteroids, topical calcineurin inhibitors, systemic agents and antibacterial medicines) during the 2‐year study period.

**Table 4:** Number and proportion of patients with at least one recorded prescription in any of the atopic dermatitis medicine groups (2017–2018)

Medicine group	Patients with atopic dermatitis (ever)^†^ (*N* = 345 742)	% of patients with atopic dermatitis (ever)	95% CI
Topical corticosteroids	126 096	36.5	35.6, 37.3
Mild	25 888	7.5	7.1, 7.9
Moderate	39 608	11.5	10.9, 12.0
Potent	77 806	22.5	21.8, 23.2
Very potent	2767	0.8	0.7, 0.9
Topical calcineurin inhibitors	250	0.1	0.0, 0.1
Systemic corticosteroids	53 944	15.6	14.9, 16.4
Systemic immunosuppressants	3001	0.9	0.8, 0.9
Antibacterial medicines^‡^	112 092	32.4	31.6, 33.2

^†^ Note that the number of patients in each medicine group and in each class of topical corticosteroids is not mutually exclusive as some patients may be prescribed medicines from more than one group and/or from more than one class of topical corticosteroids.

^‡^ Antibacterial medicines included in this analysis are mupirocin, dicloxacillin, flucloxacillin, cefalexin, clindamycin (non‐topical form), trimethoprim/sulfamethoxazole and erythromycin.

Patients with atopic dermatitis were more likely to have an eosinophil count recorded (59.5%; 95% CI 58.2–60.8) than patients without atopic dermatitis (54.2%; 95% CI 53.0–55.4). Patients with atopic dermatitis were 60% more likely to have eosinophilia compared to those without atopic dermatitis (age–sex‐adjusted relative risk 1.6; 95% CI 1.5–1.6) (data not shown).

## Discussion

This is the first large‐scale Australian study to estimate the prevalence, incidence and management of atopic dermatitis using routinely collected general practice data. Over 2.1 million regular patients, including 345 742 identified as having atopic dermatitis, were included. As atopic dermatitis is a chronic relapsing condition with some patients outgrowing the disease, we estimated both the lifetime prevalence (current or past history of atopic dermatitis) and current prevalence (atopic dermatitis recorded during the 2‐year study period), in order to provide a more comprehensive picture.

The estimated lifetime prevalence of atopic dermatitis in the Australian general practice setting was 16.4%, and the current prevalence was 6.3%. As expected, the prevalence of current atopic dermatitis was highest for younger patients but was more common for older patients than previously recognised.^24^ The high prevalence of eczema among older MedicineInsight patients could represent a true finding or an overestimate resulting from the relative incompleteness of records among younger, healthier patients compared to older patients; mortality attrition bias arising if deceased patients are less likely to have eczema recorded; and misclassification bias from the inclusion of patients with ‘other dermatitis and a family or personal history of atopy’ and historical eczema diagnoses that resolved.

A population‐based study in Melbourne estimated current prevalence (eczema symptoms in the past year) of eczema in 4‐year‐olds as 16% in 2017 and lifetime prevalence (ever‐recorded diagnosis) as 27.6%.^6^ Our study found current and lifetime prevalence of AD in patients aged 0–4 years of 13.8% and 18.8%, respectively. Two other population‐based studies in Victoria also reported estimated current prevalence of atopic dermatitis, one in school‐aged children as 16.3%^25^ and the other in adults aged 20 and over as 6.9%^26^. Both studies used a survey followed by physical examination for identifying atopic dermatitis and reported higher prevalence of mild (and minimal) atopic dermatitis, compared to our patient cohort, which may explain the higher overall prevalence in these studies compared to ours. Evidence from the International Study of Asthma and Allergies in Childhood demonstrated a wide variation in the current prevalence of eczema globally – from 0.9% in India to 22.5% in Ecuador for 6‐ to 7‐year‐olds and from 0.2% in China to 24.6% in Colombia for 13‐ to 14‐year‐olds.^2^ This variation may reflect true differences, or disparate study designs, sampling methodologies, definitions of eczema and reported age groups.

Incidence of atopic dermatitis in the general practice setting was 2.0% in 2018. The highest incidence of atopic dermatitis was in the youngest age group (0–4 years: 3.9%). Gender‐related differences were observed for some age groups, namely a higher incidence among females than males aged 5–39 and 45–54 years. This is one of the few studies to report the incidence of atopic dermatitis in Australia.

Topical corticosteroids are first‐line treatment and very effective for most cases of atopic dermatitis.^27^ Just over a third of patients with atopic dermatitis had a prescription for topical corticosteroids during the study period. Potent topical corticosteroids were the most common class of TCS and included methylprednisolone, mometasone furoate and betamethasone dipropionate. As topical calcineurin inhibitors are second‐line atopic dermatitis therapy for patients where topical corticosteroid therapy is contraindicated or has failed,^27^, ^28^, ^29^ and may be more expensive than their corresponding class of topical corticosteroids,^30^ this may explain the very small proportion of patients prescribed them during the 2‐year study. Non‐corticosteroid immunosuppressants, generally prescribed by specialists, were not surprisingly, sparsely recorded.

Guidelines recommend oral corticosteroids be used for atopic dermatitis only with careful consideration.^17^, ^28^, ^29^ Our finding that one in six patients with atopic dermatitis were prescribed oral corticosteroids is potentially concerning. Several factors might explain the relatively high use of oral corticosteroids in this cohort of atopic dermatitis patients including potential gaps in consumer and GP knowledge about optimising the use of topical therapies before moving to oral corticosteroids, and use for conditions other than atopic dermatitis.

About 40% of patients with a history of atopic dermatitis did not have a prescription for any of the specified medicine groups (topical corticosteroids, topical calcineurin inhibitors, systemic agents and antibacterial medicines) over the 2 years of the study. It is possible that some patients may have outgrown the disease; obtained their prescriptions from a non‐MedicineInsight GP or a specialist; and been managed only with over‐the‐counter therapies (such as mild topical corticosteroids or emollients) or with other therapies not assessed in this study.

We found that patients with atopic dermatitis were more likely to have eosinophilia than those without atopic dermatitis, which is similar to findings reported elsewhere.^31^

Consistent with other studies,^10^, ^11^, ^12^ our findings demonstrate that patients with atopic dermatitis were at increased risk of comorbidities, including insomnia, anxiety and depression, compared to patients with no record of atopic dermatitis. This reinforces the importance of health professionals addressing comorbidities which decrease the quality of life for patients with atopic dermatitis.^17^

The strengths of this analysis include the size and national coverage of the MedicineInsight data. As MedicineInsight is an open cohort and patients in Australia can visit multiple general practices, we used a cohort of regularly attending patients, likely to be receiving most of their care at a MedicineInsight practice, to help improve data quality. The use of clinical records reduces subjective biases found in self‐reported health surveys, because these data comprise GP‐identified diagnoses and medicines prescribed for patients. MedicineInsight has comprehensive medicines information including both PBS/RPBS‐subsidised and private (non‐subsidised) medicines.

These data have limitations, in addition to those inherent in routinely collected data described elsewhere.^19^ For privacy reasons, MedicineInsight does not include data from progress notes, which may contain further clinical information. Depending on individual GP recording practices, a diagnosis for a current condition may have only been recorded historically, and not routinely, thus underestimating current prevalence. Utilisation of medicines, particularly mild TCS (including 0.5% and 1% hydrocortisone preparations which are available over the counter), may have been underestimated. Some people may have been prescribed a relevant medication (such as oral prednisolone) for a different condition – because the reason for prescription was not commonly recorded, we could not directly link relevant prescriptions with the diagnosis of atopic dermatitis. Lastly, while our definition of ‘moderate‐to‐severe' atopic dermatitis would have identified most patients with severe atopic dermatitis, patients with moderate disease may have been underestimated if they did not use systemic therapies or have a record for referral to a specialist.

Our findings provide important insights about the epidemiology of atopic dermatitis and its management in Australian general practice. This information is useful for planning effective interventions to support GPs, and other primary care providers, in the optimal management of their patients with atopic dermatitis, particularly those experiencing uncontrolled atopic dermatitis and acute flares.

## Supporting information


**Figure S1** Flowchart for the sub‐analysis cohort and incident atopic dermatitis population.
**Table S1** Clinical definitions used to identify atopic and co‐morbid conditions.
**Table S2** Classification of topical corticosteroids by potency.
**Table S3** Socio‐demographic characteristics for patients with ‘current’ atopic dermatitis (2017–2018).
**Table S4** Socio‐demographic characteristics for the sub‐analysis cohort and patients with incident atopic dermatitis (2018).

## References

[1] Australasian Society of Clinical Immunology and Allergy . Eczema (Atopic Dermatitis). 2019 27 June 2019 (Accessed 1 July 2019); Available from: https://www.allergy.org.au/patients/skin‐allergy/eczema.

[2] Odhiambo JA , Williams HC , Clayton TO *et al* Global variations in prevalence of eczema symptoms in children from ISAAC Phase Three. J. Allergy Clin. Immunol. 2009; 124: 1251–8.e23. 20004783 10.1016/j.jaci.2009.10.009

[3] Deckers IA , McLean S , Linssen S *et al* Investigating international time trends in the incidence and prevalence of atopic eczema 1990–2010: a systematic review of epidemiological studies. PLoS ONE 2012; 7: e39803. 22808063 10.1371/journal.pone.0039803 PMC3394782

[4] Nutten S . Atopic dermatitis: global epidemiology and risk factors. Ann. Nutr. Metab. 2015; 66(Suppl 1): 8–16. 25925336 10.1159/000370220

[5] Bieber T . Atopic dermatitis. N. Engl. J. Med. 2008; 358: 1483–94. 18385500 10.1056/NEJMra074081

[6] Peters RL , Koplin JJ , Gurrin LC *et al* The prevalence of food allergy and other allergic diseases in early childhood in a population‐based study: HealthNuts age 4‐year follow‐up. J. Allergy Clin. Immunol. 2017; 140: 145–53.e8. 28514997 10.1016/j.jaci.2017.02.019

[7] Martin PE , Koplin JJ , Eckert JK *et al* The prevalence and socio‐demographic risk factors of clinical eczema in infancy: a population‐based observational study. Clin. Exp. Allergy 2013; 43: 642–51. 23711126 10.1111/cea.12092

[8] Harris VR , Cooper AJ . Atopic dermatitis: the new frontier. Med. J. Aust. 2017; 207: 351–6. 29020907 10.5694/mja17.00463

[9] Weidinger S , Novak N . Atopic dermatitis. Lancet 2016; 387: 1109–22. 26377142 10.1016/S0140-6736(15)00149-X

[10] Yaghmaie P , Koudelka CW , Simpson EL . Mental health comorbidity in patients with atopic dermatitis. J. Allergy Clin. Immunol. 2013; 131: 428–33. 23245818 10.1016/j.jaci.2012.10.041 PMC3565469

[11] Schmitt J , Buske‐Kirschbaum A , Roessner V . Is atopic disease a risk factor for attention‐deficit/hyperactivity disorder? A systematic review. Allergy 2010; 65: 1506–24. 20716320 10.1111/j.1398-9995.2010.02449.x

[12] Dalgard FJ , Gieler U , Tomas‐Aragones L *et al* The psychological burden of skin diseases: a cross‐sectional multicenter study among dermatological out‐patients in 13 European countries. J. Invest. Dermatol. 2015; 135: 984–91. 25521458 10.1038/jid.2014.530 PMC4378256

[13] Dieris‐Hirche J , Gieler U , Petrak F *et al* Suicidal ideation in adult patients with atopic dermatitis: a german cross‐sectional study. Acta Derm. Venereol. 2017; 97: 1189–95. 28676884 10.2340/00015555-2741

[14] Drucker AM , Wang AR , Li WQ *et al* The burden of atopic dermatitis: summary of a report for the national eczema association. J. Invest. Dermatol. 2017; 137: 26–30. 27616422 10.1016/j.jid.2016.07.012

[15] Adamson AS . The economics burden of atopic dermatitis. Adv. Exp. Med. Biol. 2017; 1027: 79–92. 29063433 10.1007/978-3-319-64804-0_8

[16] Mooney E , Rademaker M , Dailey R *et al* Adverse effects of topical corticosteroids in paediatric eczema: Australasian consensus statement. Australas. J. Dermatol. 2015; 56: 241–51. 25752907 10.1111/ajd.12313

[17] Smith S , Baker C , Gebauer K *et al* Atopic dermatitis in adults: An Australian management consensus. Australas. J. Dermatol. 2019; 61: 23–32. 31372984 10.1111/ajd.13124

[18] Williams H , Stewart A , von Mutius E *et al* Is eczema really on the increase worldwide? J Allergy Clin Immunol 2008; 121: 947–54.e15. 18155278 10.1016/j.jaci.2007.11.004

[19] Busingye D , Gianacas C , Pollack A *et al* Data Resource Profile: MedicineInsight, an Australian national primary health care database. Int J Epidemiol 2019 10.1093/ije/dyz147. 31292616

[20] Australasian Society of Clinical Immunology and Allergy . Laboratory Tests in the Diagnosis of Allergic Diseases. 2019 January 2010 (Accessed 17 October 2019); Available from: https://www.allergy.org.au/hp/papers/tests‐in‐the‐diagnosis‐of‐allergic‐diseases.

[21] Australian Bureau of Statistics . Australian Statistical Geography Standard (ASGS): Volume 5 ‐ Remoteness Structure, July 2016. 2018 (Accessed 7 May 2018); Available from: http://www.abs.gov.au/ausstats/abs@.nsf/Latestproducts/1270.0.55.005Main%20Features1July%202016?opendocument&tabname=Summary&prodno=1270.0.55.005&issue=July%202016&num=&view=.

[22] Australian Bureau of Statistics . Socio‐Economic Indexes for Areas. 2018 (Accessed 7 May 2018); Available from: http://www.abs.gov.au/websitedbs/censushome.nsf/home/seifa.

[23] Krzywinski M , Altman N . Points of significance: error bars. Nat. Methods 2013; 10: 921–2. 24161969 10.1038/nmeth.2659

[24] Silverberg JI . Public health burden and epidemiology of atopic dermatitis. Dermatol. Clin. 2017; 35: 283–9. 28577797 10.1016/j.det.2017.02.002

[25] Marks R , Kilkenny M , Plunkett A *et al* The prevalence of common skin conditions in Australian school students: 2. Atopic dermatitis. Br J Dermatol 1999; 140: 468–73. 10233268 10.1046/j.1365-2133.1999.02711.x

[26] Plunkett A , Merlin K , Gill D *et al* The frequency of common nonmalignant skin conditions in adults in central Victoria, Australia. Int. J. Dermatol. 1999; 38: 901–8. 10632768 10.1046/j.1365-4362.1999.00856.x

[27] Eichenfield LF , Tom WL , Berger TG *et al* Guidelines of care for the management of atopic dermatitis: section 2. Management and treatment of atopic dermatitis with topical therapies. J. Am. Acad. Dermatol. 2014; 71: 116–32. 24813302 10.1016/j.jaad.2014.03.023 PMC4326095

[28] Rubel D , Thirumoorthy T , Soebaryo RW *et al* Consensus guidelines for the management of atopic dermatitis: an Asia‐Pacific perspective. J. Dermatol. 2013; 40: 160–71. 23289827 10.1111/1346-8138.12065

[29] Wollenberg A , Barbarot S , Bieber T *et al* Consensus‐based European guidelines for treatment of atopic eczema (atopic dermatitis) in adults and children: part I. J. Eur. Acad. Dermatol. Venereol. 2018; 32: 657–82. 29676534 10.1111/jdv.14891

[30] Strathie Page S , Weston S , Loh R . Atopic dermatitis in children. Aust. Fam. Physician 2016; 45: 293–6. 27166464

[31] Jenerowicz D , Czarnecka‐Operacz M , Silny W . Peripheral blood eosinophilia in atopic dermatitis. Acta Dermatovenerol. Alp. Pannonica Adriat. 2007; 16: 47–52. 17992457

