# Supplementary material for: Prevalence, incidence and management of atopic dermatitis in Australian general practice using routinely collected data from MedicineInsight
Source: Australas J Dermatol. 2020 Mar 15;61(3):e319–27. doi: 10.1111/ajd.13268 (PMC7496939; doi:10.1111/ajd.13268)
Supplement: Supplementary file 1 — Figure S1 Flowchart for the sub‐analysis cohort and incident atopic dermatitis population. Table S1 Clinical definitions used to identify atopic and co‐morbid conditions. Table S2 Classification of topical corticosteroids by potency. Table S3 Socio‐demographic characteristics for patients with ‘current’ atopic dermatitis (2017–2018). Table S4 Socio‐demographic characteristics for the sub‐analysis cohort and patients with incident atopic dermatitis (2018). [file AJD-61-e319-s001.docx]

**Appendix**

**Figure S1** Flowchart for the sub-analysis cohort and incident atopic dermatitis population

**Main study population**

Patients with at least 3 clinical encounters during the 2-year study and with valid information for age and sex

(**2,104,909**)

Patients with at least 12 months of follow-up (attendance) at the practice prior to 1 January 2018

(**1,637,549**)

Patients without 12 months of follow-up at the practice prior to 1 January 2018

(**467,360**)

**Sub-analysis cohort**

Patients without a diagnosis of atopic dermatitis prior to 1 January 2018

(**1,349,224**)

Patients with a diagnosis of atopic dermatitis prior to 1 January 2018

(**288,325**)

**Incident atopic dermatitis cohort**

Patients with a new diagnosis of atopic dermatitis in 2018

(**27,414**)

Patients without atopic dermatitis

(**1,321,810**)

**Table S1** Clinical definitions used to identify atopic and co-morbid conditions

| **Condition** | **Definition** |
| --- | --- |
| Atopic dermatitis | Patients were defined as having atopic dermatitis if they had a relevant coded (from Docle or Pyefinch) or free text entry in one of the three diagnosis fields recorded at any time from the patient's earliest record up to the date of the end of the study. Relevant terms included: atopic dermatitis. |
| Eczema | Patients were defined as having eczema if they had a relevant coded (from Docle or Pyefinch) or free text entry in one of the three diagnosis fields recorded at any time from the patient's earliest record up to the date of the end of the study. Relevant terms included: eczema (not including seborrheic, senile pruritus). |
| Dermatitis - other | Patients were defined as having other dermatitis if they had a relevant coded (from Docle or Pyefinch) or free text entry in one of the three diagnosis fields recorded at any time from the patient's earliest record up to the date of the end of the study. Relevant terms included: dermatitis (not including seborrheic/cradle cap, senile pruritus). |
| Atopy | Patients were defined as having atopy if they had a relevant coded (from Docle or Pyefinch) or free text entry in one of the three diagnosis fields recorded at any time from the patient's earliest record up to the date of the end of the study. Relevant terms included: atopy, allergic disorder or allergic diathesis. |
| Asthma | Patients were defined as having asthma if they had a relevant coded (from Docle or Pyefinch) or free text entry in one of the three diagnosis fields recorded at any time from the patient's earliest record up to the date of the end of the study. Relevant terms included: asthma or asthma care plan |
| Allergic rhinoconjunctivitis (includes allergic rhinitis or conjunctivitis separately or in combination) | Patients were defined as having allergic rhinoconjunctivitis if they had a relevant coded (from Docle or Pyefinch) or free text entry in one of the three diagnosis fields recorded at any time from the patient's earliest record up to the date of the end of the study. Relevant terms included: allergic rhinitis, allergic conjunctivitis, allergic rhinoconjunctivitis, hay fever, pollen allergy, seasonal rhinitis or vernal conjunctivitis. |
| Food allergies (e.g., milk, eggs, tree nuts or peanuts, sesame, fish and seafood) | Patients were defined as having food allergy if they had a relevant coded (from Docle or Pyefinch) or free text entry in one of the three diagnosis fields recorded at any time from the patient's earliest record up to the date of the end of the study. Relevant food allergy terms included: allergy, hypersensitivity or anaphylactic shock/reaction to milk (dairy), eggs, nuts (tree or peanuts), sesame, fish and seafood |
| Insomnia | Patients were defined as having insomnia if they had a relevant coded (from Docle or Pyefinch) or free text entry in one of the three diagnosis fields recorded at any time from the patient's earliest record up to the date of the end of the study. Relevant terms included: insomnia, sleep disturbance, difficulty sleeping or poor sleep |
| Anxiety | Patients were defined as having anxiety if they had a relevant coded (Docle, Pyefinch) or free text entry in one of the three diagnosis fields recorded at any time from the patient's earliest record up to the date of the end of the study. Relevant terms included: anxiety, anxiety/depression; anxiety disorder, depressive anxiety disorder, GAD, generalised anxiety disorder, mixed anxiety/depressive disorder, nervous anxiety, neurotic anxiety, social anxiety disorder, social phobia |
| Depression | Patients were defined as having depression if they had a relevant coded (from Docle or Pyefinch) or free text entry in one of the three diagnosis fields recorded at any time from the patient's earliest record up to the date of the end of the study. Relevant terms included: depression (all types), depressive anxiety disorder, anxiety/depression, mixed anxiety depression, mixed anxiety/depressive disorder or adjustment disorder |

**Table S2** Classification of topical corticosteroids by potency

| **Potency** | **Topical corticosteroid** | **Strength** | **Preparations** | **Example indications** |
| --- | --- | --- | --- | --- |
| **Mild (class I)** | hydrocortisone | 0.5%, 1% | cream, ointment, spray | facial and flexural dermatitis |
|  | hydrocortisone acetate | 0.5%, 1% | cream, ointment |  |
| **Moderate**  **(class II)** | betamethasone valerate | 0.02% | cream | mild-to-moderate atopic dermatitis |
|  | betamethasone valerate | 0.05% | cream, ointment |  |
|  | clobetasone butyrate | 0.05% | cream |  |
|  | desonide | 0.05% | lotion |  |
|  | triamcinolone acetonide | 0.02% | cream, ointment |  |
| **Potent (class III)** | betamethasone dipropionate | 0.05% | cream, ointment, lotion | short-term use in severe inflammatory dermatoses |
|  | betamethasone valerate | 0.1% | cream, ointment |  |
|  | methylprednisolone aceponate | 0.1% | cream, ointment, lotion |  |
|  | mometasone furoate | 0.1% | cream, ointment, hydrogel, lotion |  |
| **Very potent (class IV)** | betamethasone dipropionate | 0.05% (in an optimised vehicle) | cream, ointment | severe eczema, also useful for eczema of hands and feet |
|  | clobetasol propionate* | 0.05% | cream, ointment, lotion, shampoo |  |

*Clobetasol propionate as a cream, ointment or lotion is not registered for use in Australia, but is available via the [Special Access Scheme](https://tgldcdp.tg.org.au/viewTopic?topicfile=drug-availability-in-palliative-care&sectionId=pcg4-c28-s3#pcg4-c28-s3) or compounding pharmacies. Only use under expert supervision. Table adapted from Australian Medicines Handbook. Eczema: Drugs for eczema, <https://amhonline.amh.net.au/chapters/dermatological-drugs/tables/topical-corticosteroids-table> (accessed 5 July 2019).

**Table S3** Socio-demographic characteristics for patients with ‘current’ atopic dermatitis (2017–2018)

| Characteristic | | Patients with current atopic dermatitis (N = 133,085) | |
| --- | --- | --- | --- |
|  |  | Number | % (95% CI) |
| **Sex** | |  |  |
|  | Male | 55,576 | 41.76 (41.21, 42.31) |
|  | Female | 77,509 | 58.24 (57.69, 58.79) |
| **Age group (years)** | |  |  |
|  | 0–4 | 19,910 | 14.96 (13.96, 15.96) |
|  | 5–9 | 9,098 | 6.84 (6.54, 7.14) |
|  | 10–14 | 5,838 | 4.39 (4.18, 4.60) |
|  | 15–19 | 5,818 | 4.37 (4.20, 4.55) |
|  | 20–24 | 6,675 | 5.02 (4.72, 5.31) |
|  | 25–29 | 6,725 | 5.05 (4.75, 5.35) |
|  | 30–34 | 7,134 | 5.36 (5.03, 5.69) |
|  | 35–39 | 6,982 | 5.25 (4.98, 5.51) |
|  | 40–44 | 6,297 | 4.73 (4.55, 4.92) |
|  | 45–49 | 6,532 | 4.91 (4.75, 5.07) |
|  | 50–54 | 6,558 | 4.93 (4.74, 5.11) |
|  | 55–59 | 7,397 | 5.56 (5.32, 5.79) |
|  | 60–64 | 7,148 | 5.37 (5.12, 5.62) |
|  | 65–69 | 7,491 | 5.63 (5.31, 5.94) |
|  | 70–74 | 7,455 | 5.60 (5.24, 5.97) |
|  | 75–79 | 6,117 | 4.60 (4.25, 4.94) |
|  | 80–84 | 4,774 | 3.59 (3.30, 3.87) |
|  | 85–89 | 3,056 | 2.30 (2.09, 2.50) |
|  | 90+ | 2,080 | 1.56 (1.40, 1.72) |
| **State/territory** | |  |  |
|  | ACT | 2,843 | 2.14 (0.43, 3.84) |
|  | NSW | 51,095 | 38.39 (32.35, 44.43) |
|  | NT | 1,332 | 1.00 (0.20, 1.80) |
|  | QLD | 17,869 | 13.43 (9.98, 16.87) |
|  | SA | 3,771 | 2.83 (1.19, 4.48) |
|  | TAS | 9,078 | 6.82 (3.90, 9.75) |
|  | VIC | 34,095 | 25.62 (19.44, 31.79) |
|  | WA | 13,002 | 9.77 (6.33, 13.21) |
| **Remoteness** | |  |  |
|  | Major city | 89,263 | 67.07 (61.83, 72.31) |
|  | Inner regional | 28,120 | 21.13 (17.01, 25.25) |
|  | Outer regional | 14,092 | 10.59 (6.97, 14.21) |
|  | Remote/very remote | 1,610 | 1.21 (0.53, 1.89) |
| **Socio-economic status (SEIFA IRSAD quintiles)** | |  |  |
|  | 1 (least advantaged) | 21,355 | 16.05 (12.61, 19.49) |
|  | 2 | 23,057 | 17.33 (14.30, 20.35) |
|  | 3 | 29,729 | 22.34 (19.22, 25.46) |
|  | 4 | 28,856 | 21.68 (18.77, 24.60) |
|  | 5 (most advantaged) | 30,019 | 22.56 (18.69, 26.42) |
|  | Missing | 69 | 0.05 (0.00, 0.12) |

CI: confidence interval; IRSAD: Index of Relative Socio-economic Advantage and Disadvantage; SEIFA: Socio-economic Indexes for Areas

**Table S4** Socio-demographic characteristics for the sub-analysis cohort and patients with incident atopic dermatitis (2018)

| Characteristic | | Sub-analysis cohort (N = 1,349,224) | | Patients with incident atopic dermatitis (N = 27,414) | |
| --- | --- | --- | --- | --- | --- |
|  |  | Number | % (95% CI) | Number | % (95% CI) |
| **Sex** | |  |  |  |  |
|  | Male | 592,491 | 43.91 (43.44, 44.38) | 11,079 | 40.41 (39.64, 41.19) |
|  | Female | 756,733 | 56.09 (55.62, 56.56) | 16,335 | 59.59 (58.81, 60.36) |
| **Age group (years)** | |  |  |  |  |
|  | 0–4 | 51,828 | 3.84 (3.66, 4.02) | 2,017 | 7.36 (6.85, 7.87) |
|  | 5–9 | 67,623 | 5.01 (4.82, 5.20) | 1,727 | 6.30 (5.92, 6.68) |
|  | 10–14 | 58,001 | 4.30 (4.14, 4.46) | 1,250 | 4.56 (4.24, 4.88) |
|  | 15–19 | 64,222 | 4.76 (4.61, 4.91) | 1,311 | 4.78 (4.48, 5.08) |
|  | 20–24 | 68,658 | 5.09 (4.85, 5.33) | 1,309 | 4.77 (4.42, 5.13) |
|  | 25–29 | 72,959 | 5.41 (5.11, 5.70) | 1,288 | 4.70 (4.38, 5.02) |
|  | 30–34 | 84,554 | 6.27 (5.95, 6.58) | 1,535 | 5.60 (5.13, 6.07) |
|  | 35–39 | 90,546 | 6.71 (6.44, 6.99) | 1,642 | 5.99 (5.60, 6.38) |
|  | 40–44 | 88,823 | 6.58 (6.41, 6.76) | 1,560 | 5.69 (5.34, 6.04) |
|  | 45–49 | 100,092 | 7.42 (7.27, 7.56) | 1,628 | 5.94 (5.65, 6.23) |
|  | 50–54 | 95,017 | 7.04 (6.92, 7.16) | 1,581 | 5.77 (5.45, 6.09) |
|  | 55–59 | 100,594 | 7.46 (7.30, 7.61) | 1,842 | 6.72 (6.36, 7.08) |
|  | 60–64 | 94,523 | 7.01 (6.81, 7.20) | 1,765 | 6.44 (6.08, 6.80) |
|  | 65–69 | 89,736 | 6.65 (6.39, 6.91) | 1,837 | 6.70 (6.28, 7.12) |
|  | 70–74 | 80,413 | 5.96 (5.66, 6.26) | 1,744 | 6.36 (5.91, 6.82) |
|  | 75–79 | 57,053 | 4.23 (3.98, 4.47) | 1,388 | 5.06 (4.63, 5.49) |
|  | 80–84 | 40,061 | 2.97 (2.78, 3.16) | 1,048 | 3.82 (3.48, 4.16) |
|  | 85–89 | 26,156 | 1.94 (1.81, 2.07) | 600 | 2.19 (1.96, 2.41) |
|  | 90+ | 18,365 | 1.36 (1.26, 1.47) | 342 | 1.25 (1.08, 1.41) |
| **State/territory** | |  |  |  |  |
|  | ACT | 24,450 | 1.81 (0.31, 3.32) | 513 | 1.87 (0.39, 3.35) |
|  | NSW | 528,114 | 39.14 (33.64, 44.65) | 10,615 | 38.72 (32.69, 44.75) |
|  | NT | 21,842 | 1.62 (0.42, 2.82) | 365 | 1.33 (0.29, 2.37) |
|  | QLD | 212,186 | 15.73 (12.02, 19.43) | 3,871 | 14.12 (10.52, 17.73) |
|  | SA | 35,713 | 2.65 (1.10, 4.19) | 702 | 2.56 (1.06, 4.07) |
|  | TAS | 87,620 | 6.49 (3.65, 9.34) | 1,920 | 7.00 (3.93, 10.08) |
|  | VIC | 283,199 | 20.99 (16.10, 25.88) | 6,586 | 24.02 (18.03, 30.02) |
|  | WA | 156,100 | 11.57 (7.53, 15.61) | 2,842 | 10.37 (6.64, 14.09) |
| **Remoteness** | |  |  |  |  |
|  | Major city | 836,281 | 61.98 (56.85, 67.11) | 17,829 | 65.04 (59.60, 70.47) |
|  | Inner regional | 331,692 | 24.58 (20.30, 28.87) | 6,194 | 22.59 (18.17, 27.02) |
|  | Outer regional | 160,326 | 11.88 (8.56, 15.21) | 3,033 | 11.06 (7.46, 14.67) |
|  | Remote/very remote | 20,925 | 1.55 (0.71, 2.39) | 358 | 1.31 (0.60, 2.01) |
| **Socio-economic status (SEIFA IRSAD quintiles)** | |  |  |  |  |
|  | 1 (least advantaged) | 212,417 | 15.74 (12.59, 18.89) | 4,403 | 16.06 (12.67, 19.45) |
|  | 2 | 247,063 | 18.31 (15.40, 21.22) | 5,107 | 18.63 (15.33, 21.92) |
|  | 3 | 305,055 | 22.61 (19.40, 25.82) | 6,232 | 22.73 (19.39, 26.07) |
|  | 4 | 285,086 | 21.13 (18.46, 23.80) | 5,684 | 20.73 (17.86, 23.61) |
|  | 5 (most advantaged) | 298,872 | 22.15 (18.59, 25.71) | 5,973 | 21.79 (17.99, 25.59) |
|  | Missing | 731 | 0.05 (0.00, 0.12) | 15 | 0.05 (0.00, 0.13) |

CI: confidence interval; IRSAD: Index of Relative Socio-economic Advantage and Disadvantage; SEIFA: Socio-economic Indexes for Areas
